# Importance of operator training and rest perfusion on the diagnostic accuracy of stress perfusion cardiovascular magnetic resonance

**DOI:** 10.1186/s12968-018-0493-4

**Published:** 2018-11-19

**Authors:** Adriana D. M. Villa, Laura Corsinovi, Ioannis Ntalas, Xenios Milidonis, Cian Scannell, Gabriella Di Giovine, Nicholas Child, Catarina Ferreira, Muhummad Sohaib Nazir, Julia Karady, Esmeralda Eshja, Viola De Francesco, Nuno Bettencourt, Andreas Schuster, Tevfik F. Ismail, Reza Razavi, Amedeo Chiribiri

**Affiliations:** 1 School of Biomedical Engineering & Imaging Sciences, King’s College London, King’s Health Partners, 4th Floor Lambeth Wing, St Thomas’ Hospital, London, SE1 7EH UK; 2 Cardiology Department of the Basingstoke and North Hampshire Hospital, Basingstoke, UK; 3 Cardiology Department, St. Thomas’ Hospital, Guy’s and St Thomas’ NHS Foundation Trust, London, UK; 4 Faculdade de Ciências da Saúde CICS, UBI, Covilhã, Portugal; 5 Radiology Department, ICS Maugeri IRCCS, Porto, Italy; 6 Cardiovascular R&D Unit, Faculty of Medicine, University of Porto, Porto, Portugal; 7 Department of Cardiology, Royal North Shore Hospital, The Kolling Institute, Northern Clinical School, University of Sydney, Sydney, Australia; 8 Department of Cardiology and Pneumology, University Medical Center Göttingen, Georg-August University, Göttingen, Germany; 9 German Center for Cardiovascular Research (DZHK), Partner Site Göttingen, Göttingen, Germany

**Keywords:** Cardiovascular magnetic resonance, Stress perfusion imaging, Coronary artery disease, Quantitative assessment, Myocardial ischemia, Diagnostic accuracy, Training

## Abstract

**Background:**

Clinical evaluation of stress perfusion cardiovascular magnetic resonance (CMR) is currently based on visual assessment and has shown high diagnostic accuracy in previous clinical trials, when performed by expert readers or core laboratories. However, these results may not be generalizable to clinical practice, particularly when less experienced readers are concerned. Other factors, such as the level of training, the extent of ischemia, and image quality could affect the diagnostic accuracy. Moreover, the role of rest images has not been clarified.

The aim of this study was to assess the diagnostic accuracy of visual assessment for operators with different levels of training and the additional value of rest perfusion imaging, and to compare visual assessment and automated quantitative analysis in the assessment of coronary artery disease (CAD).

**Methods:**

We evaluated 53 patients with known or suspected CAD referred for stress-perfusion CMR. Nine operators (equally divided in 3 levels of competency) blindly reviewed each case twice with a 2-week interval, in a randomised order, with and without rest images. Semi-automated Fermi deconvolution was used for quantitative analysis and estimation of myocardial perfusion reserve as the ratio of stress to rest perfusion estimates.

**Results:**

Level-3 operators correctly identified significant CAD in 83.6% of the cases. This percentage dropped to 65.7% for Level-2 operators and to 55.7% for Level-1 operators (*p* < 0.001). Quantitative analysis correctly identified CAD in 86.3% of the cases and was non-inferior to expert readers (*p* = 0.56). When rest images were available, a significantly higher level of confidence was reported (*p* = 0.022), but no significant differences in diagnostic accuracy were measured (*p* = 0.34).

**Conclusions:**

Our study demonstrates that the level of training is the main determinant of the diagnostic accuracy in the identification of CAD. Level-3 operators performed at levels comparable with the results from clinical trials. Rest images did not significantly improve diagnostic accuracy, but contributed to higher confidence in the results. Automated quantitative analysis performed similarly to level-3 operators. This is of increasing relevance as recent technical advances in image reconstruction and analysis techniques are likely to permit the clinical translation of robust and fully automated quantitative analysis into routine clinical practice.

## Background

Stress perfusion cardiovascular magnetic resonance (CMR) is increasingly used for the evaluation of patients with known or suspected coronary artery disease (CAD) and has a class I indication for patients at intermediate risk of CAD according to recent guidelines [1, 2].

Stress perfusion CMR has been shown to be highly accurate for the detection of CAD, with sensitivity ranging from 75 to 91% and specificity ranging from 59 to 87% [3–5]. It should be noted that in most of these studies, visual assessment has been carried out either by a core laboratory or by expert readers, and therefore the findings may not be generalizable to routine clinical practice. As stress perfusion CMR gains acceptance and becomes more available, it will inevitably be performed in lower volume and less experienced centers.

Stress perfusion CMR is typically evaluated by visual assessment. This can be influenced by the extent of ischemia and the presence of areas of relatively preserved perfusion, which can be used as reference [6]. Moreover, image artefacts can complicate the interpretation of the images. Dark rim artefacts, which are commonly observed during stress perfusion, can be misdiagnosed as subendocardial perfusion abnormalities [7], in particular when relatively long acquisition times are used and spatial resolution is low. Moreover, areas of infarction are frequently associated with delayed perfusion [8, 9]. The simultaneous evaluation of stress and rest perfusion CMR and late gadolinium enhancement (LGE) images is recommended to identify areas of myocardial infarction and improve the specificity of the interpretation [10, 11], and to exclude imaging artefacts [10].

Additionally, it has been suggested that rest perfusion images could play an important role in improving the identification of imaging artefacts when signal abnormalities are present on both stress and rest images [10]. The acquisition of rest images enables quantification of perfusion reserve, but prolongs scan times and requires additional contrast dosing.

Stress perfusion CMR is complex to read and requires significant training and experience. However, the impact of training and experience has not been formally studied and as yet, there are no specific recommendations in current guidelines, apart from stating that stress perfusion CMR should be part of the training program for Level-2 readers [12]. It is hoped that fully quantitative automated methods may help bridge training gaps and support clinical decision making.

We sought to determine the importance of the level of training of the operator on the diagnostic accuracy of stress perfusion CMR; the role of rest perfusion images in the identification of imaging artefacts and in the correct detection of CAD; and to systematically compare the results of visual assessment with semi-automated quantitative analysis to determine its additional value.

## Methods

Consecutive patients (*n* = 53) referred for stress perfusion CMR for suspected CAD were retrospectively included in the study. All patients had invasive coronary angiography on the basis of the clinical indication within 1 month of the CMR examination. Exclusion criteria were contraindications to CMR, gadolinium-based contrast agents or adenosine. Patients with previous coronary artery bypass grafting, hypertrophic cardiomyopathy, aortic stenosis, or other primary myopathic or valvular disease were excluded. All subjects gave written informed consent in accordance with ethical approval. This study complies with the Declaration of Helsinki.

### Image acquisition

CMR images were acquired using a 3T scanner (Achieva, Philips Healthcare, Best, The Netherlands) equipped with 32-channel phased-array cardiac coil. The protocol included functional assessment, adenosine stress and rest first pass perfusion imaging, and LGE. The images were acquired using standard acquisition protocols and in end-expiratory breath-hold. For stress imaging, 140 μg/kg/min of adenosine was administered. Imaging commenced at least 3 minutes after infusion initiation. A dual bolus (equal volumes of 0.0075 mmol/kg followed by 0.075 mmol/kg after a 20-s pause) of contrast agent (gadobutrol/Gadovist, Schering, Germany) was injected at 4 ml/s by a power injector [13]. For perfusion, a saturation recovery prepared gradient echo pulse sequence accelerated with k–t sensitivity encoding acceleration with 11 training profiles was used. Typical imaging parameters were: 3 short-axis slices covering standard American Heart Association (AHA) segments [14], 120 acquired dynamics/slice, flip angle 20°, TR 2.5 ms, TE 1.25 ms, saturation pre-pulse recovery time 100 ms, pixel size 1.9 × 1.9 mm, slice thickness 10 mm.

Typical imaging parameters for LGE imaging were: long and short axis to fully cover the left ventricle, inversion recovery turbo field echo, flip angle 25°, TR 6 ms, TE 3 ms, pixel size 0.7 × 0.7 mm, slice thickness 10 mm.

### Operator selection

Nine operators were chosen amongst the physicians working in our unit and in other European institutions, on the basis of their level of competency, according to the European Society of Cardiology (ESC)/European Association of Cardiovascular Imaging (EACVI) training guidelines [12]. A total of 9 operators, 3 for each competency level, were chosen; all operators had recently obtained the ESC/EACVI certification (within 2 months) for the appropriate level. In brief, level-1 competency ESC certification requires 20 continuous medical education (CME) hours, involvement in 50 CMR cases and 1-month fellowship; level-2 requires at least 50 CME hours, involvement in 150 clinical cases of which 25 must be perfusion studies, a minimum of 3-months fellowship and the European CMR exam; level-3 requires at least 50 CME hours, involvement in 300 clinical cases of which a minimum of 50 must be perfusion studies, at least 12-months training and the European CMR exam. Level-1 competency reflects core CMR training, level-2 is required to report CMR studies with support from a Level-3 operator and Level-3 is required to perform, interpret and report CMR studies fully independently [12].

### Image analysis – Visual assessment

**Fig. 1:**
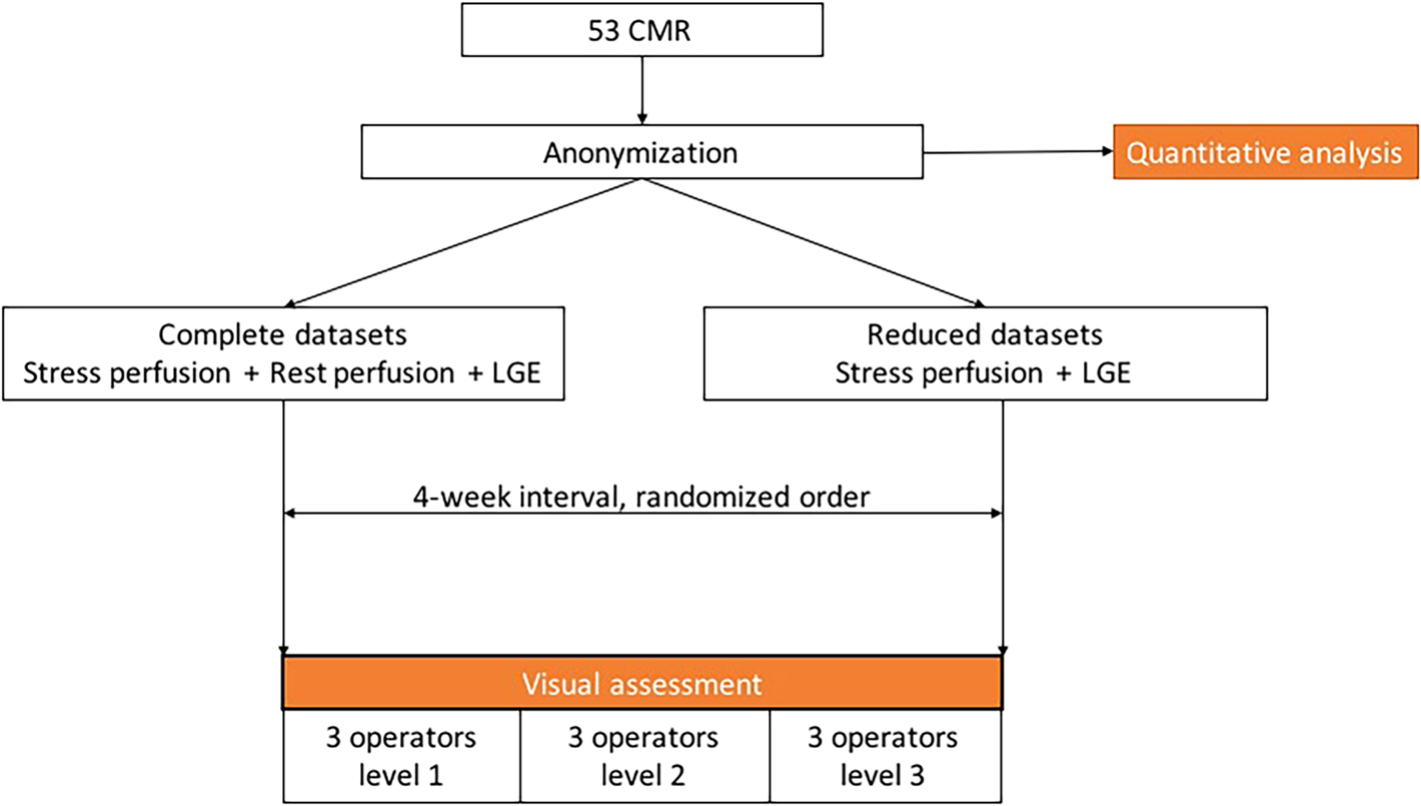
Study flowchart. CMR: cardiovascular magnetic resonance, LGE: late gadolinium enhancement

Each operator was asked to report each of the 53 scans twice over a 4-week period, with a minimum interval of 2 weeks between first and second read. The scans were anonymized and presented to the operator as a full dataset, including stress and rest perfusion and LGE, or as reduced datasets, including stress perfusion and LGE only. The full and reduced datasets were analysed blinded to clinical and angiographic data and in a randomized order on different days. The study flowchart can be seen in Fig. 1.

Visual assessment of adenosine stress perfusion CMR and LGE images, displayed side-by-side, was performed as per clinical practice, in accordance with standardized CMR protocols [15]. A perfusion defect was defined as a regional reduction in myocardial signal during LV first-pass of contrast agent, not related to artefacts and not corresponding to an area of scar on LGE images.

Operators were asked to fill an on-line standardized form and to identify segments with inducible ischemia, to identify the presence and transmurality of LGE [16], to identify the most likely culprit coronary artery based on the standard AHA segmentation [14], and to grade their confidence in the diagnosis and the perceived image quality.

The confidence was graded as: 0- very unconfident, 1- unconfident, 2- confident, 3- very confident. The perceived image quality was graded as: 0- poor, 1- moderate, 2- good, 3- excellent.

Coronary angiography results have been used as reference standard. The threshold for coronary artery lumen stenosis was 70% diameter stenosis for epicardial vessels. All invasive angiographic images have been reviewed by consensus of expert operators.

### Image analysis – Quantitative assessment

A different operator, blinded to results of visual perfusion assessment and other clinical/angiographic data, performed the segmentation of the images for semi-automated quantitative analysis using software and methods previously developed and validated by our group. Respiratory motion was corrected using affine image registration by maximization of the joint correlation between consecutive dynamics within an automatically determined region of interest [17]. A temporal maximum intensity projection was calculated to serve as a feature image for automatic contour delineation method. The operator then manually optimized the automatically generated contours to avoid partial volume effects at the endocardial and epicardial borders [17]. The intervention of the operator was limited to image segmentation. Quantitative perfusion analysis was then automatically performed by Fermi-constrained deconvolution according to the methods described by Wilke et al. [18] and Jerosch-Herold et al. [19], optimised for high-resolution pixel-wise analysis [20, 21]. Myocardial perfusion reserve (MPR) was calculated as the ratio between stress and rest myocardial blood flow (MBF) estimates. Ischemia was defined as segments with MPR < 1.5, according to previously validated criteria [22, 23].

### Statistical analysis

Continuous variables are presented as mean ± standard deviation for normally distributed variables and as median with interquartile range for non-parametric data. Normality was assessed with Q-Q plots and the Kolmogorov-Smirnov test. Continuous variables were compared using an unpaired Student *t test* or the Wilcoxon rank-sum test, as appropriate, and categorical data were compared between groups using the Fisher exact test and Pearson chi-square test. The McNemar test was used for paired dichotomous data. Two-tailed values of *p* < 0.05 were considered to be statistically significant. One-way ANOVA was used to determine differences between multiple groups. Bonferroni correction was used to account for multiple testing.

## Results

### Characteristics of the population

The mean age of the population (*n* = 53) was 60.6 ± 12.7 years. Demographic data are shown in Table 1. The prevalence of CAD in the group of patients included in the analysis was 30.2%, with 16/53 patients positive for CAD on invasive coronary angiography. Left anterior descending (LAD) lesions were identified in 9 (17%) of the cases, left circumflex (LCX) lesions in 8 (15.1%) of the cases, and right coronary artery (RCA) in 13 (24.5%) of the cases. Within the group of patients with CAD, 8 patients had 1-vessel disease (50%), 5 patients 2-vessel disease (31.3%) and 3 patients 3-vessel disease (18.8%).

**Table 1:** Demographic characteristics of the population

	All (*n* = 53)
Age (years)	60.6 ± 12.7
Male gender	36 (67.9%)
Hypertension	30 (56.6%)
Dyslipidaemia	23 (43.4%)
Diabetes	10 (18.9%)
Current smoker	13 (24.5%)
Previous PCI	9 (17%)
Family history of CAD	12 (22.6%)

*PCI* percutaneous coronary artery intervention, *CAD* coronary artery disease

### Impact of operator training on correct CAD identification

**Fig. 2:**
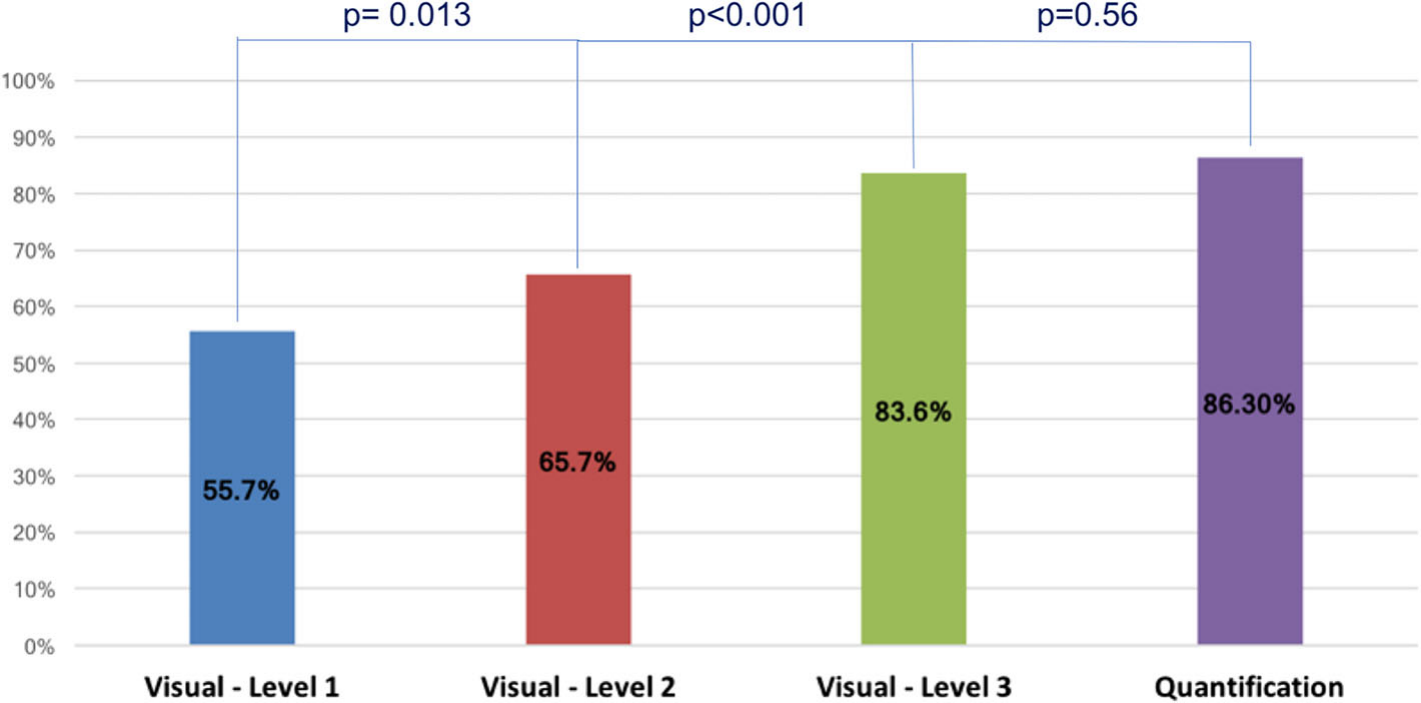
Percentage of correct coronary artery disease (CAD) identification (diagnostic accuracy) for different levels of CMR training and using quantitative assessment. CAD: coronary artery disease, CMR: cardiovascular magnetic resonance

**Fig. 3:**
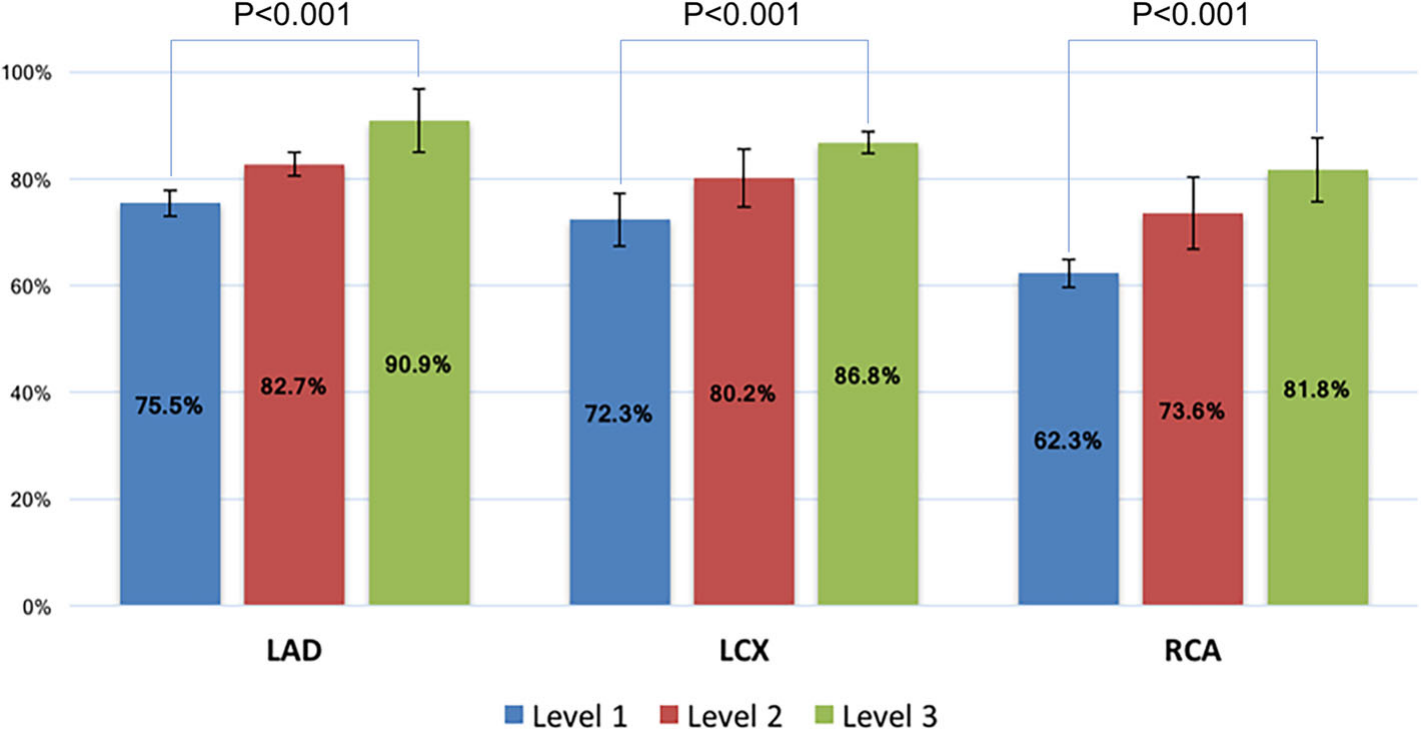
Percentage of correct CAD identification (diagnostic accuracy) stratified by coronary territory. CAD: coronary artery disease, LAD: left anterior descending coronary artery, LCX: left circumflex coronary artery, RCA: right coronary artery

There was a significant correlation between an operator’s training level and the rate of correct identification of CAD on a per patient level on visual assessment. The diagnosis of Level-3 operators agreed with invasive coronary angiography in 83.6 ± 2.3% of the cases, while this percentage dropped to 65.7 ± 4.3% for Level-2 operators and to 55.7 ± 5.3% for Level-1 operators (*p* < 0.001 between the 3 groups) (Fig. 2). A significant difference in the agreement with angiography between different levels of training was also observed in a sub-analysis per coronary territory (p < 0.001) (Fig. 3). When different perfusion territories were compared, the agreement between CMR and coronary angiography was higher for the LAD territory, followed by the LCX and by the RCA territories. The same trend was observed in all groups of operators, regardless of the level of training (p < 0.001).

**Fig. 4:**
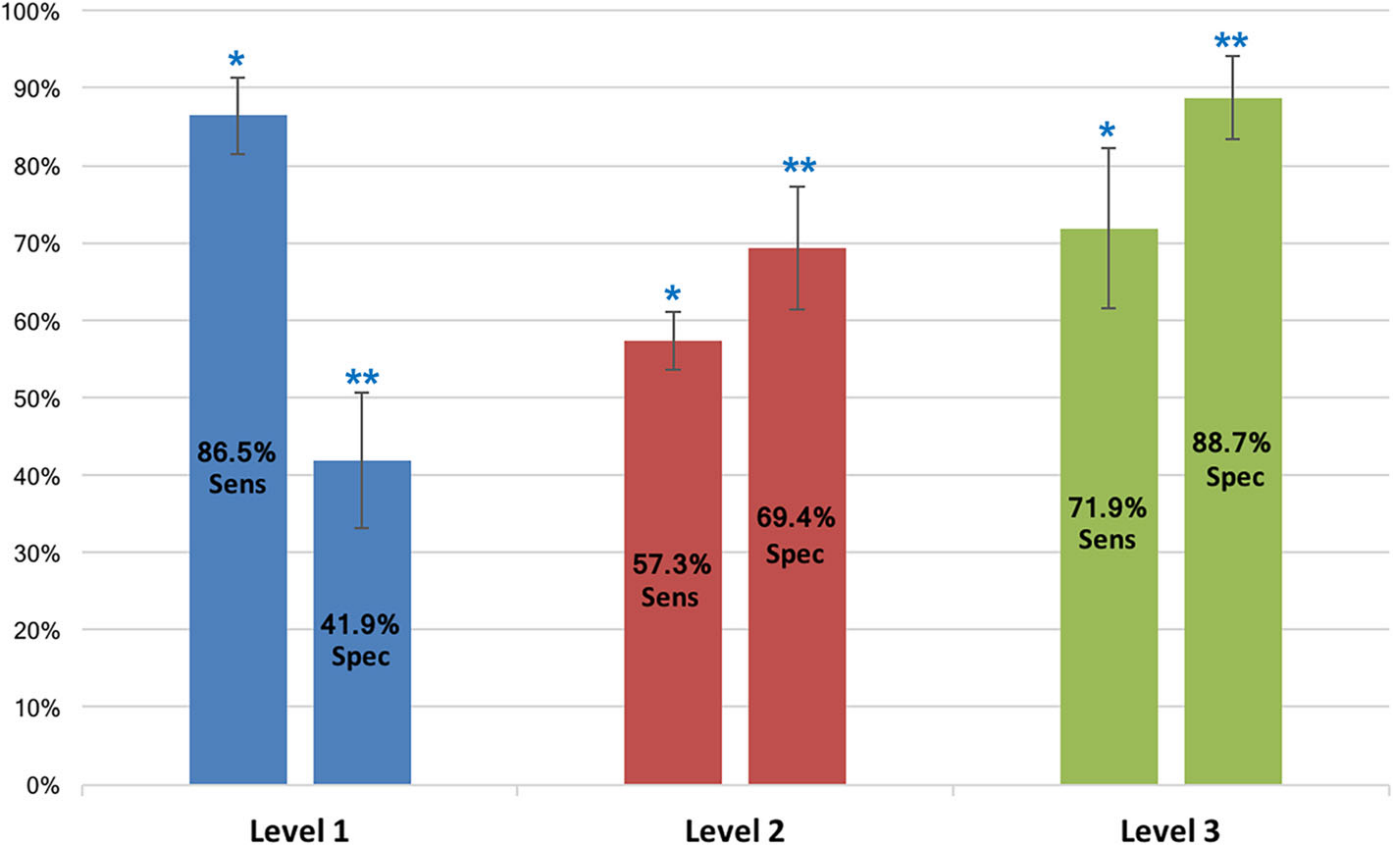
Sensitivity and specificity for level of CMR training. * denotes statistically significant difference (*p* < 0.001) between sensitivity values. ** denotes statistically significant difference (*p* < 0.001) between specificity values. Sens: sensitivity, spec: specificity

The sensitivity and specificity for operators of different levels of training are reported in Fig. 4. Level-1 operators showed high sensitivity (86.5 ± 6.1%) and low specificity (41.9 ± 10.9%). Level-2 operators had a sensitivity of 57.3 ± 4.7% and a specificity of 69.4 ± 9.9%. Level-3 operators showed a sensitivity of 71.9 ± 13% and a specificity of 88.7 ± 6.7% respectively. There was a statistically significant difference for both sensitivity and specificity between different levels of training (p < 0.001) (Fig. 4).

### Impact of rest perfusion on correct identification of CAD

**Fig. 5:**
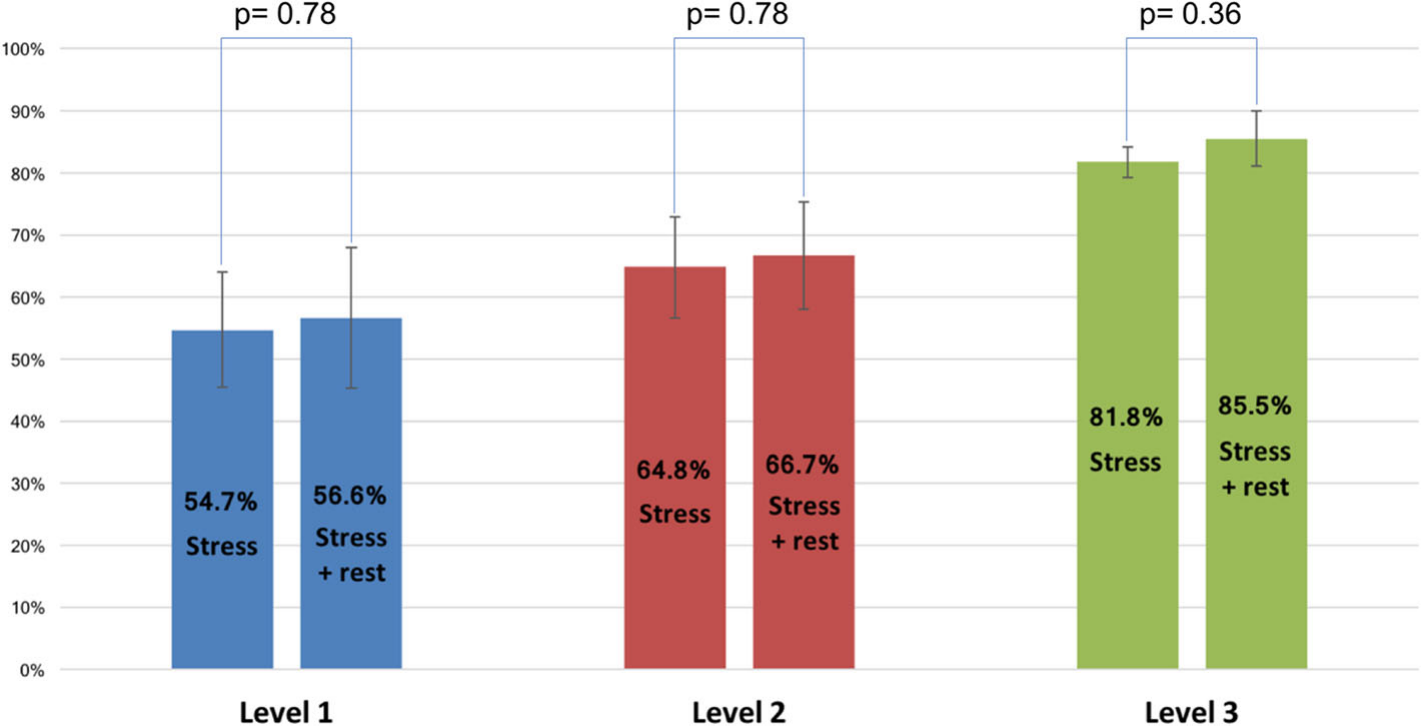
Percentage of correct identification of CAD (diagnostic accuracy) using stress perfusion only or stress and rest images. CAD: coronary artery disease

When rest images were available, there was no statistically significant difference at all levels of training (Fig. 5) and in the overall analysis (69.6 ± 14.3% vs 67.1 ± 13.1%; *p* = 0.34). However, when rest images were available, a significantly higher level of confidence was reported by the operators (*p* = 0.022) and subjective image quality was scored at a higher level (*p* = 0.012).

### CAD classification

**Fig. 6:**
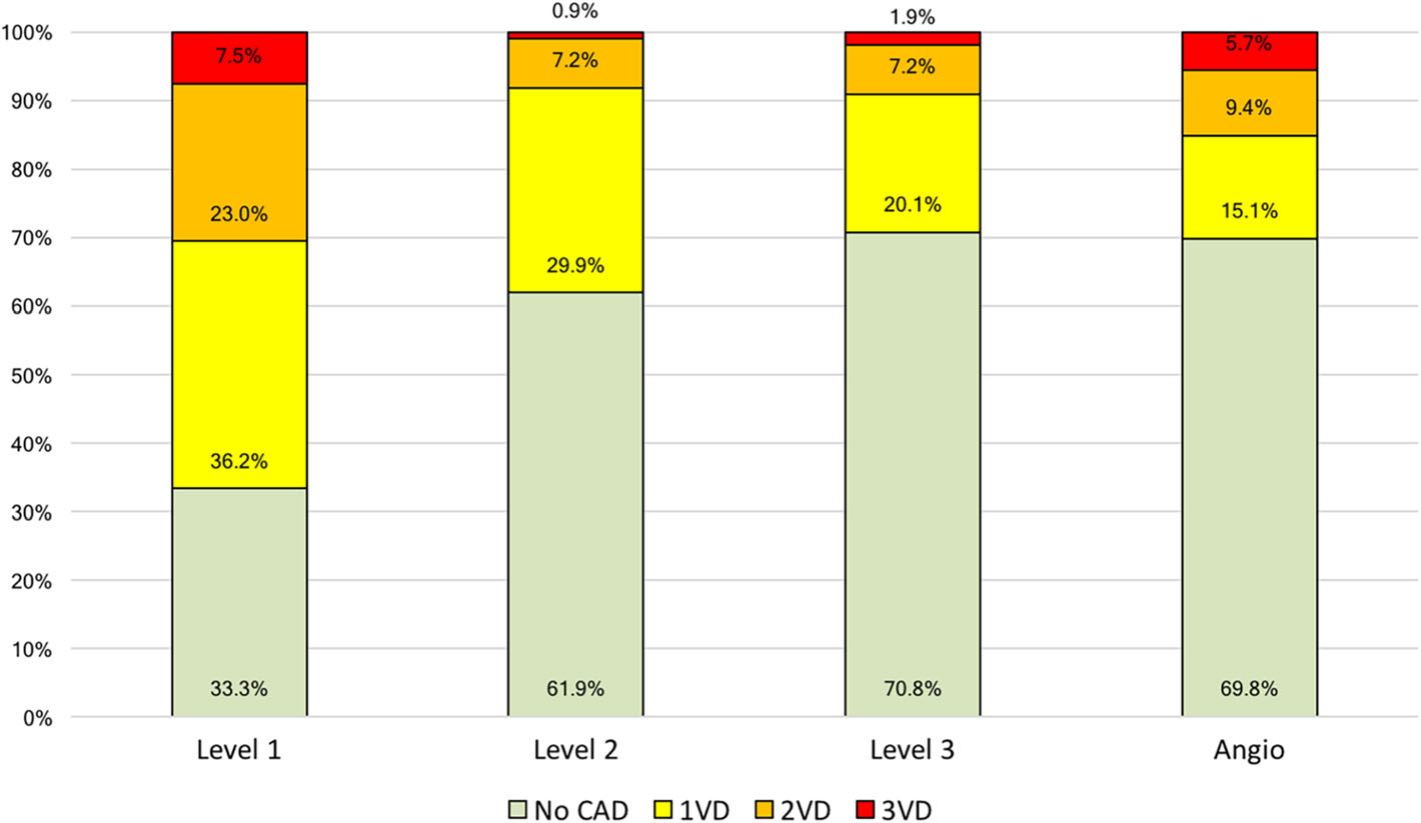
CAD classification for different levels of CMR training. CAD: coronary artery disease, 1VD: one-vessel disease, 2VD, two-vessel disease, 3VD: three-vessel disease

Figure 6 shows a comparison between the extent of CAD identified by the operators on CMR images in comparison with invasive coronary angiography. An overestimation of the severity of CAD was observed in Level-1 operators, regardless of the number of vessels with CAD. Despite being more accurate, Level-2 and Level-3 operators significantly underestimated the number of positive perfusion territories in patients with multi-vessel CAD.

### Impact of quantitative analysis on correct CAD identification

Quantitative analysis was successfully performed in 51 patients. In 2 cases of patients without CAD, the automated algorithms failed and no results could be calculated. In both cases, this was due to the low quality of the diluted pre-bolus used for the estimation of the arterial input function. Level-3 visual assessment of the 2 cases where quantification failed yielded the correct diagnosis in both cases when both stress and rest images were made available to the readers, and in 66% of interpretations when only stress perfusion was made available to the readers. Quantitative stress perfusion CMR analysis agreed with the results of invasive angiography in 86.3% of the cases, performing significantly better than Level-1 and Level-2 operators (*p* < 0.001). Level-3 visual assessment and quantitative analysis were not significantly different (*p* = 0.56) (Fig. 2). Quantitative analysis had a sensitivity of 68.8% and specificity of 94.3%. When the 2 cases in which quantitative analysis failed are considered as a missed diagnosis, the concordance of quantitative analysis with invasive angiography was 83%, with a sensitivity of 68.8% and a specificity of 89.2%.

## Discussion

This study has several important findings. Operator training and experience had a significant impact on diagnostic accuracy. Only Level-3 trained operators had an accuracy comparable with the results reported by large clinical trials [3–5]. Rest images did not significantly improve the diagnostic accuracy of stress perfusion CMR but, when available, contributed to a significantly higher confidence of the operators in their reports and to a higher perceived image quality, regardless of the level of training. Finally, semi-automated quantitative analysis performed better than Level-1 and Level-2 operators, but similarly to a Level-3 operator. Quantitative analysis however failed in 2/53 cases due to technical reasons related to the administration of the diluted pre-bolus. However, the same cases could be analysed visually.

Stress perfusion CMR plays an increasingly important role in the evaluation of patients with known or suspected CAD. Recent European guidelines recommend the use of stress perfusion CMR in patients with suspected CAD and intermediate pre-test probability, with a class 1 indication and level of evidence A, similarly to stress echocardiography and nuclear imaging [1, 2]. US guidelines recommend stress perfusion CMR with 2A indication [24], particularly in specific subgroups of patients [25]. These indications are based on the assumption that stress perfusion CMR is highly accurate for the identification of CAD and compares favorably with other functional modalities. In large trials and meta-analyses, the sensitivity ranged from 75% [3] to 91% [4] and specificity ranged from 59% [3] to 87% [5]. In the CE-MARC study [26], sensitivity was 86.5% and specificity was 83.4%, and the MR-IMPACT 2 trial [27] reported a sensitivity of 75% and a specificity of 59%. These wide intervals most likely represent the variability in study design, the different prevalence of disease in different populations, and variability in the criteria used for visual assessment.

The diagnostic accuracy of stress perfusion CMR reported in the literature is often the result of visual assessment carried out by expert readers, which are usually Level-3 operators and often are internationally recognized experts.

Our study demonstrates that the diagnostic accuracy varied significantly amongst groups of readers with different levels of training, and reached values comparable with those of large studies only in the group of Level-3 operators. These results confirm the high diagnostic accuracy of stress perfusion CMR in comparison with coronary angiography, however clearly indicate the need for Level-3 supervision when stress perfusion scans are reported.

From the analysis of the sensitivity and specificity for the detection of CAD in different groups, it emerges that Level-1 operators had high sensitivity (86.5%). This came however at the cost of a reduced specificity (41.9%) and rate of overall correct CAD detection (55.7%). Factors such as image quality and the prevalence of dark rim artefacts, which can mimic the presence of subendocardial perfusion defects, could have played a role. In comparison, Level-3 operators under-called the disease (sensitivity 71.9%), but had a high specificity (88.7%). All diagnostic investigations involve a trade-off between sensitivity and specificity. At a population level and from a health-economic perspective, we feel that the results achieved by Level 3 operators represent a reasonable balance between the need to identify significant coronary disease and the high specificity required to avoid increasing down-stream investigation costs through increased referral for invasive coronary angiography. The work of Patel et al. [28] highlights the need for better selection of patients for invasive investigation given the costs and potential morbidity incurred by this.

Our results support the recommendations from the ESC [12], which state that Level-1 operators hold the basic knowledge in CMR sufficient to select appropriate CMR indications and interpret CMR reports, but are not cleared to report CMR scans. This is reflected in our result by the fact that Level-1 operators demonstrated a very low diagnostic accuracy, with poor specificity for the presence of CAD. According to the ESC guidelines, Level-2 operators may actively perform and report CMR, but are not completely independent and should work under the supervision of a Level-3 expert. This is also supported by our results, since Level-2 operators were significantly less accurate than Level-3 operators. Level-3 operators instead performed to levels similar to those reported by studies such as the CE-MARC [26].

It should be noted that the Society for Cardiovascular Magnetic Resonance (SCMR) guidelines on training [29] differ slightly from the ESC guidelines used in this study to define the level of training of the operators. According to the SCMR guidelines, Level-2 operators can independently report CMR scans, whereas Level-3 certification has more to do with being able to lead a CMR unit and perform research in the field. Both guidelines agree that Level-1 training is not sufficient to practice CMR.

It has been suggested that rest perfusion images play an important role in improving the identification of imaging artefacts when signal abnormalities are present on both stress and rest images [10]. When assessing stress perfusion CMR visually, guidelines advise displaying both rest and stress images side-by-side to identify correctly inducible perfusion defect and artefacts [10, 11].

In our study, we did not find any significant difference in the diagnostic accuracy when rest images were available. Our findings mirror those of Biglands et al. [30]. However, when testing the operator confidence and the perceived image quality, a statistically significant difference was noted when both stress and rest images were available. The increased confidence was more evident for Level-1 and Level-2 operators.

Interestingly, Level-1 operators reported a higher confidence score than more experienced operators, despite lower overall accuracy. This could reflect a cognitive bias, also known as the Dunning-Kruger effect [31].

The diagnostic usefulness of rest perfusion imaging resides in the finding of “fixed perfusion defect” on both stress and rest images, which may be related to artefacts or to areas of myocardial infarction. However, this may be overcome when stress perfusion CMR is assessed visually side-by-side with LGE, as per guidelines [11] and as in our study. Nevertheless, rest perfusion imaging remains a fundamental requirement for perfusion quantification and MPR estimation.

Semi-automated quantitative assessment performed better than Level-1 and Level-2 operators and similarly to Level-3 operators for the detection of CAD. The latter is in keeping with the results of several other studies that reported high sensitivity and specificity for quantitative analysis, with sensitivity ranging from 80% [22] to 94.4% [32] and specificity ranging from 81% [33] to 100% [34]. Previous studies from Patel et al. [6] and Mordini et al. [35] compared quantitative with visual and semi-quantitative analysis and demonstrated that quantitative analysis is superior to visual assessment and semi-quantitative assessment in the detection of ischemia, and that quantitative analysis is the most accurate method to measure the total ischemic burden.

In the present study, quantitative analysis was performed using a semi-automated method which requires user input to confirm the automated segmentation of the images, but eliminates inter-observer variability for what concerns the quantification procedure. This is of increasing relevance as recent technical advances in image reconstruction and analysis techniques are likely to permit the clinical translation of robust and fully automated quantitative analysis into routine clinical practice [36–39]. In our study however, the dual bolus approach used for arterial input function measurements failed in 2 subjects, impeding quantitative analysis. The advent of dual sequences capable of a more accurate assessment of the concentration of gadolinium in the main bolus input function may make the use of dual bolus redundant in the near future [37, 40].

### Limitations

This study included a selected population with suspected CAD and we excluded patients with primary cardiomyopathy. Thus, our results on diagnostic accuracy do not include other patterns of perfusion abnormalities, which may require even more experience to discern (e.g., microvascular dysfunction).

Moreover, we used an anatomical reference standard (invasive coronary angiography) to compare operators’ performances in interpreting a functional test, while a functional reference standard (e.g., fractional flow reserve) may be more appropriate.

Our results demonstrate that similarly accurate detection of CAD can be achieved by Level-3 operators and by automated perfusion quantification. Although our study was not powered to demonstrate the superiority of quantitative analysis, this has been the subject of a recent study which has reported very similar findings [30]. The non-inferiority of automated quantification to expert visual reads, in combination with the prognostic value of quantitative analysis [23] will facilitate more widespread adoption of stress perfusion CMR by less experienced readers.

Finally, all stress perfusion CMR were acquired in a single center, using a 3 T Philips scanner and a high-resolution k-t sequence. This may not reflect the standard clinical acquisition in other centres.

## Conclusions

This study demonstrates that visual assessment of stress perfusion CMR is challenging for Level-1 and Level-2 operators but accurate in the hands of Level-3 operators. Our results highlight the importance of the recommendations of the ESC/EACVI training guidelines in CMR, which recommend independent reporting for Level-3 operators only and supervised reporting for Level-2 trained operators. The availability of rest perfusion images was associated with significantly higher confidence and higher perceived image quality, regardless of the level of training of the operator. Quantitative analysis performed similarly to Level-3 trained operators and could represent, in the future, a valid alternative to visual assessment.

## Abbreviations

AHA: American Heart Association
CAD: Coronary artery disease
CME: Continuous medical education
CMR: Cardiovascular magnetic resonance
EACVI: European Association of Cardiovascular Imaging
ESC: European Society of Cardiology
LAD: Left anterior descending coronary artery
LCX: Left circumflex coronary artery
LGE: Late gadolinium enhancement
MBF: Myocardial blood flow
MPR: Myocardial perfusion reserve
RCA: Right coronary artery
SCMR: Society for Cardiovascular Magnetic Resonance
